# Temporal relationship between sleep duration and obesity among Chinese Han people and ethnic minorities

**DOI:** 10.1186/s12889-023-15413-4

**Published:** 2023-03-15

**Authors:** Zhengxing Xu, Min Chen, Yuntong Yao, Lisha Yu, Peijing Yan, Huijie Cui, Ping Li, Jiaqiang Liao, Ben Zhang, Yuqin Yao, Zhenmi Liu, Xia Jiang, Tao Liu, Chenghan Xiao

**Affiliations:** 1 Department of Epidemiology and Biostatistics, Institute of Systems Epidemiology, West China School of Public Health and West China Fourth Hospital, West China-PUMC C. C. Chen Institute of Health, Sichuan University, Chengdu, Sichuan China; 2 Guizhou Province Centre for Disease Control and Prevention, 101 Bageyan Road, Yunyan District, 550004 Guiyang, Guizhou Province China; 3 Department of Hygienic Toxicology, West China School of Public Health and West China Fourth Hospital, Sichuan University, 610041 Chengdu, China; 4 Department of Maternal, Child and Adolescent Health, West China School of Public Health and West China Fourth Hospital, Sichuan University, No.16, Section 3, Renmin Nan Lu, 610041 Chengdu, Sichuan China; 5 Department of Nutrition and Food Hygiene, West China School of Public Health and West China Fourth Hospital, Sichuan University, 610041 Chengdu, China

**Keywords:** Temporal relationship, Sleep duration, Obesity, Han people, Ethnic minorities

## Abstract

**Background:**

No studies have assessed the association between sleep duration and obesity in Chinese ethnic minorities. Whether the relationship between sleep duration and obesity is different between Chinese Han people and Chinese ethnic minorities remains unclear. The study aimed to explore the relationship between sleep duration and obesity among Chinese Han people and Chinese ethnic minorities.

**Methods:**

We applied data from the Guizhou Population Health Cohort Study (GPHCS), which 9,280 participants were recruited in the baseline survey from 2010 to 2012, and 8,163 completed the follow-up survey from 2016 to 2020. A total of 5,096 participants (3,188 Han Chinese and 1,908 ethnic minorities) were included in the ultimate analysis. Information on sleep duration (total 24-hour sleep time), body mass index (BMI), and waist circumference (WC) was collected at the baseline and follow-up survey, respectively. Cross-lagged panel analyses were conducted to explore the temporal relationship between sleep duration and obesity for Han people and ethnic minorities.

**Results:**

For Han people, the results from cross-lagged panel analyses indicated that baseline sleep duration was significantly associated with follow-up BMI (β_BMI_ = -0.041, 95% CI_BMI_: -0.072 ~ -0.009) and follow-up WC (β_WC_ = -0.070, 95%CI_WC_: -0.103 ~ -0.038), but baseline BMI (β_BMI_ = -0.016, 95% CI_BMI_: -0.050 ~ 0.018) and baseline WC (β_WC_ = -0.019, 95% CI_WC_: -0.053 ~ 0.016) were not associated with follow-up sleep duration. In addition, the relationship between baseline sleep duration and follow-up BMI was gender-specific and significant only in the Han people female (β_BMI_ = -0.047, 95% CI_BMI_: -0.090 ~ -0.003) but not in the Han people male (β_BMI_ = -0.029, 95% CI_BMI_: -0.075 ~ 0.016). For ethnic minorities, the results indicated that there was no relationship between sleep duration and obesity at all, either from sleep duration to obesity (β_BMI_ = 0.028, 95%CI_BMI_: -0.012 ~ 0.068; β_WC_ = 0.020, 95%CI_WC_: -0.022 ~ 0.062), or from obesity to sleep duration (β_BMI_ = -0.022, 95%CI_BMI_: -0.067 ~ 0.022; β_WC_ = -0.042, 95%CI_WC_: -0.087 ~ 0.003).

**Conclusion:**

The relationship pattern between sleep duration and obesity across Han people and ethnic minorities is different. Future sleep-aimed overweight and obesity intervention should be conducted according to population characteristics.

**Supplementary Information:**

The online version contains supplementary material available at 10.1186/s12889-023-15413-4.

## Background

Obesity has become a global epidemic and a significant health challenge worldwide [1]. There were more than 650 million adults suffering from obesity, according to the world health organization report [2]. Thus, a growing body of studies has been done to identify modifiable risk factors for obesity.

In the recent two decades, sleep duration, a critical measurement reflecting sleep quality, has been proposed as a potential factor contributing to obesity [3]. In general, most existing evidence consented to the association between short sleep duration and obesity in adults [4–8]. However, some evidence indicated that the effect size and direction of this association might vary across populations and countries [9–12]. For findings about effect size, one study suggested that African Americans with short sleep duration are more susceptible to obesity than Caucasians [9]. Furthermore, for findings about the association direction, though most studies indicated a unidirectional inversely effect from sleep duration to obesity [4–6], some studies from western developed countries indicated different association patterns [10–12]. For instance, studies from the United States and Britain consented to a unidirectional inversely effect from obesity to sleep duration [10, 11], while a study from the Netherlands indicated a bidirectional inversely relationship between obesity and sleep duration [12]. One explanation for the inconsistent findings is that some sociodemographic factors may moderate the effect size and direction of the association between sleep duration and obesity. Unfortunately, existing evidence mainly comes from western developed countries. We know little if the relationship between sleep duration and obesity will vary among people in developing countries.

With regard to developing countries, China has been suffering a surge of obesity in the past decades [13]. About 85 million Chinese adults with body mass index (BMI) ≥ 28.0 kg/m² in 2018, the figure was three times compared with 2004 [14]. Furthermore, China is a unified multi-ethnic country consisting of Han people and 55 ethnic minorities, of which the minority population exceeds 125 million [15]. Sleep duration and obesity are significantly different among Han and ethnic minorities in China due to variations in sociodemographic factors [16, 17]. However, it remains unclear whether the relationship between sleep duration and obesity is different between Han people and ethnic minorities. When exploring the association between sleep duration and obesity in China, most available studies only focused on Chinese Han people [18, 19], leading to a poor understanding of the association for Chinese ethnic minorities. For the paucity of previous ethnic minorities studies, one possible reason is the inadequacy of minority samples. Although ethnic minorities account for 8.89% of China’s total population [15], most sample surveys fail to obtain sufficient representative samples for valid statistical inference due to widespread distribution across the country [20]. Guizhou province is located in southwestern China, it is one of the primary concentrations of ethnic minorities in China, with more than 36.44% of the whole province’s population being ethnic minorities, including the Miao, Buyi, Dong and so forth [21]. The extensive minority population in Guizhou province offers the possibility to explore the relationship between sleep duration and obesity among ethnic minorities.

Therefore, leveraging a longitudinal data from Guizhou containing a significant proportion of Chinese ethnic minorities (37.44%), this study attempted to examine the relationship between sleep duration and obesity across Chinese Han people and ethnic minorities. Given the evidence from relevant studies in western developed countries [10–12] and also considering that Chinese Han people and ethnic minorities have different demographic characteristics [16, 17], this study hypothesized that the relationship between sleep duration and obesity is different among Chinese Han people and ethnic minorities. This study can contribute to the knowledge about the association between sleep duration and obesity, and the moderating effect of ethnicity on the relationship among Chinese people.

## Methods

### Study population and sample

We used data from two stages of the Guizhou Population Health Cohort Study (GPHCS) to accomplish the analyses. The GPHCS conducted a multistage stratified cluster random sampling method to recruit participants in Guizhou province, China. Detailed information related to study design and sampling strategy has been reported elsewhere [22]. Briefly, a total of 9,280 individuals aged 18 years and older from 48 townships of 12 districts in Guizhou province were recruited from November 2010 to December 2012, and 8,163 individuals completed the follow-up survey from December 2016 to June 2020. This study was approved by the Institutional Review Board of Guizhou Province Centre for Disease Control and Prevention (No. S2017-02) [22]. All participants signed informed consent before the data collection.

**Fig. 1:**
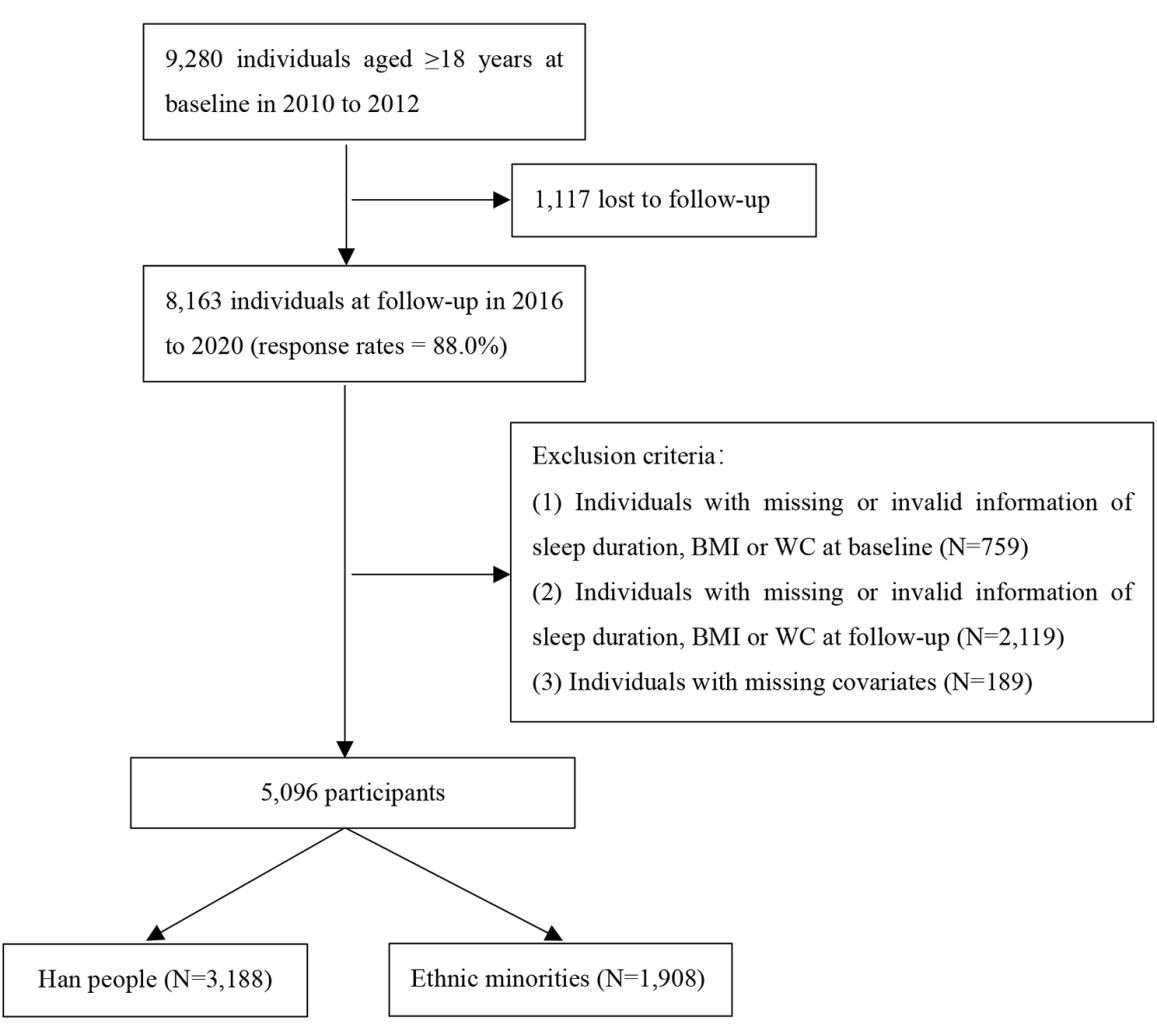
Flow chart of participants

To explore the relationship between sleep duration and obesity, we excluded 3,067 individuals with missing or invalid information for sleep duration, height, weight, waist circumference, or other covariates (e.g., drinking, energy intake, or physical activity). At last, we included a total of 5,096 participants in the subsequent analysis, with an average follow-up period of 7.12 years (standard deviation = 1.13 years) (Fig. 1).

### Assessment of sleep duration

Self-reported sleep duration was obtained through a questionnaire by asking “how long do you sleep on a typical day?“. It’s important to note that the sleep duration assessed in this study refers to the 24-hour total sleep time. Each participant’s answer was converted to hours to represent the total sleep duration per day.

### Anthropometric measurements

Anthropometric measurements, including height, weight, and waist circumference (WC), were obtained by a standardized physical examination. Height and weight were measured using unified height meters (accuracy is 0.1 cm) and electronic weight scales (accuracy is 0.1 kg). Body mass index (BMI) was calculated as weight in kilograms (kg) divided by height in meters squared (m^2^). WC (cm) was measured using a waist ruler (accuracy is 0.1 cm) at the midpoint between the lower rib cage and the iliac crest.

### Measurement of covariates

Information on several covariates, including age, gender, place of residence, education levels, marital status, smoking status, alcohol consumption, dietary energy intake, physical activity, and sedentary behavior, were collected to adjust for confounding factors. Place of residence was categorized as 1 = rural and 2 = urban. Education was classified into four categories: 1 = illiterate, 2 = primary school, 3 = secondary school, and 4 = college and above. Marital status was classed as 1 = married and 2 = other (separation, divorce, widowed, spinsterhood, or cohabit). Smoking status was categorized as 1 = non-smoker and 2 = smoker. Drinking status was categorized as 1 = non-drinker and 2 = drinker. Energy intake (kcal/day) was assessed by daily dietary intake. Specifically, habitual diets regarding the previous 12 months were assessed using a semi-quantitative food frequency questionnaire (FFQ) with 14 food groups (cereals, tubers, pork, livestock, poultry, aquatic products, vegetables, fruits, juice and beverage, eggs, dairy products, bean products, and fried products) [23]. For each food group, participants were required to report the quantity and frequency. According to the information from FFQ, we estimated the total daily energy intake based on the Chinese Food Composition Tables published in 2009 [24]. Physical activity and sedentary behavior were assessed using the Global Physical Activity Questionnaire [25], and physical activity intensity level was classified as 1 = low, 2 = moderate, and 3 = high [26].

### Statistical analyses

Analyses were performed by using R version 4.0.5. All tests were conducted on two-sided, and *P* value less than or equal to 0.05 was considered statistically significant.

**Fig. 2:**
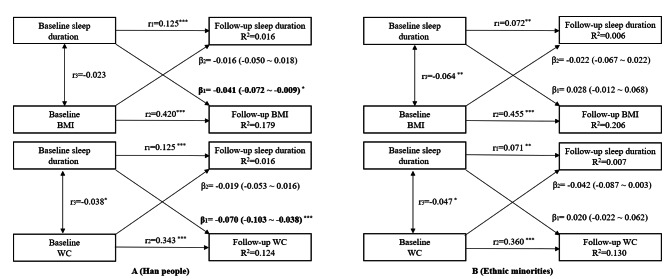
Cross-lagged path analysis of sleep duration with BMI and WC in the Han people (A) and ethnic minorities (B), adjusted for age, gender, place of residence, education levels, marital status, smoking status, alcohol consumption, dietary energy intake, physical activity, sedentary behavior, and follow-up years; β_1_ represents cross-lagged path coefficients from baseline sleep duration to follow-up BMI or WC; β_2_ represents from baseline BMI or WC to follow-up sleep duration; r_1_ and r_2_ represent tracking correlations; r_3_ represent synchronous correlations; R^2^ represents variance explained. Goodness-of-fit (A, Han people, BMI): CFI = 1, RMR = 0.004; Goodness-of-fit (A, Han people, WC): CFI = 0.994, RMR = 0.010; Goodness-of-fit (B, Ethnic minorities, BMI): CFI = 1, RMR = 0.007; Goodness-of-fit (B, Ethnic minorities, WC): CFI = 0.996, RMR = 0.010. The cross-lagged path coefficients are presented as β (lower 95% CI, upper 95% CI). ^*^P ≤ 0.05, ^**^P ≤ 0.01, ^***^P ≤ 0.001

Cross-lagged panel analyses were performed to examine the longitudinal relationship of sleep duration with BMI and WC across Han people and ethnic minorities. The cross-lagged panel analysis is a form of path analysis that simultaneously examines reciprocal, longitudinal relationships among a set of intercorrelated variables [27], which has been widely used in epidemiological studies [11, 28, 29]. A parsimonious model version is depicted in Fig. 2. A significant path coefficient (β_1_ or β_2_) suggests the directionality between the two variables measured over time. The cross-lagged path models were estimated based on the correlation matrix using the maximum likelihood method by the R package “*Lavaan*“ [30]. The validity of model fitting was assessed by root mean square residual (RMR) and comparative fit index (CFI) [31]. RMR < 0.05 and CFI > 0.90 indicate a relatively good fit for the observed data [28, 29].

Before cross-lagged path analysis, the baseline and follow-up of sleep duration, BMI and WC were adjusted for all covariates mentioned above by regression residual analyses. Then, the residual was standardized with Z-transformation (mean = 0; standard deviation = 1) [28, 29] in Han people and ethnic minorities, respectively. At last, the standard Z score of sleep duration, BMI and WC were applied in the cross-lagged path analyses.

In addition, two sensitivity analyses were carried out in this study. First, the bootstrap simulation with 1000 replicates was performed to obtain 95% confidence intervals (CIs) to evaluate sensitivity to the distributions of cross-lagged path coefficients. The second analysis was executed by gender subgroups in Han people and ethnic minorities to test whether gender influences the temporal relationship of sleep duration with BMI and WC.

## Results

### Characteristics of study participants

As shown in Tables 1, a total of 3,188 Han people participants and 1,908 ethnic minorities participants were included in the analyses. There was a significant difference between Han people and ethnic minorities in almost all characteristics, except for age and gender. In addition, from baseline to follow-up, compared with Han people, ethnic minorities experienced a more significant reduction in sleep duration (Han people: 0.21 h, Ethnic minority: 0.31 h, *P* = 0.049) but a lesser increase in BMI (Han people: 0.69 kg/m^2^, Ethnic minority: 0.57 kg/m^2^, *P* = 0.207) and WC (Han people: 5.96 cm, Ethnic minority: 5.23 cm, *P* = 0.013).

**Table 1:** Characteristics at baseline and follow-up by Han people and Ethnic minorities

Characteristics		Total (N = 5,096)	Han people (N = 3,188)	Ethnic minorities (N = 1,908)	*P-*value
**Baseline**					
Age, years		44.02 ± 14.95	44.33 ± 15.08	43.49 ± 14.72	0.053
Male		2,425 (47.57)	1,492 (46.80)	933 (48.90)	0.147
Urban		1,796 (35.24)	1,607 (50.41)	189 (9.91)	< 0.001
Education					< 0.001
Illiteracy		1,036 (20.33)	602 (18.89)	434 (22.75)	
Primary		1,734 (34.03)	1,013 (31.78)	721 (37.79)	
Secondary		2,050 (40.23)	1,366 (42.85)	684 (35.85)	
Collage and above		276 (5.42)	207 (6.49)	69 (3.62)	
Married		4,099 (80.44)	2,608 (81.81)	1,491 (78.15)	0.001
Smoking		1,480 (29.04)	1,002 (31.43)	478 (25.05)	< 0.001
Drinking		1,694 (33.24)	980 (30.74)	714 (37.42)	< 0.001
Energy intake, kcal/d		2,112.09 ± 855.57	2,030.76 ± 826.38	2,247.98 ± 885.94	< 0.001
Physical activity					< 0.001
Low		1,289 (25.29)	743 (23.31)	546 (28.62)	
Moderate		1,126 (22.10)	790 (24.78)	336 (17.61)	
High		2,681 (52.61)	1,655 (51.91)	1,026 (53.77)	
Sedentary duration, h		4.09 ± 2.25	4.28 ± 2.31	3.77 ± 2.10	< 0.001
Sleep duration, h		7.88 ± 1.14	7.79 ± 1.17	8.05 ± 1.07	< 0.001
BMI, kg/m^2^		22.86 ± 3.10	22.99 ± 3.11	22.64 ± 3.06	< 0.001
WC, cm		76.74 ± 9.27	77.33 ± 9.33	75.77 ± 9.08	< 0.001
**Follow up**					
Sleep duration, h		7.64 ± 1.48	7.58 ± 1.55	7.74 ± 1.34	< 0.001
BMI, kg/m^2^		23.51 ± 3.25	23.68 ± 3.28	23.21 ± 3.18	< 0.001
WC, cm		82.43 ± 9.31	83.29 ± 9.33	81.00 ± 9.10	< 0.001
**Change from baseline to follow up**					
Δ Sleep duration, h		-0.25 ± 1.75	-0.21 ± 1.18	-0.31 ± 1.63	0.049
Δ BMI, kg/m^2^		0.64 ± 3.37	0.69 ± 3.45	0.57 ± 3.24	0.207
Δ WC, cm		5.69 ± 10.33	5.96 ± 10.45	5.23 ± 10.13	0.013

Abbreviation: BMI = body mass index, WC = waist circumference

Δ represents the mean change from baseline to follow-up measurements

Data are frequency (%) for categorical variables and mean ± standard deviation for continuous variables

### Cross-lagged panel analyses between sleep duration and obesity

As shown in Fig. 2, results from Cross-lagged panel analyses indicated that the relationship pattern between sleep duration and obesity across Han people and ethnic minorities could be distinct. For Han people (Fig. 2A), results indicated that baseline sleep duration was significantly associated with follow-up BMI (β_BMI_ = -0.041, 95%CI_BMI_: -0.072 ~ -0.009), and WC (β_WC_ = -0.070, 95%CI_WC_: -0.103 ~ -0.038). However, our results rejected the inverse effect from either baseline BMI (β_BMI_ = -0.016, 95%CI_BMI_: -0.050 ~ 0.018) nor baseline WC (β_WC_ = -0.019, 95%CI_WC_: -0.053 ~ 0.016) to follow-up sleep duration. Model fitting parameters (RMR = 0.004 and CFI = 1 in the Sleep duration-BMI model and RMR = 0.010 and CFI = 0.994 in the Sleep duration-WC model) indicated an acceptable model fitness.

Distinct from Han people, our results indicated that there was no relationship between sleep duration and obesity for ethnic minorities at all (Fig. 2B). The significant relationship between baseline sleep duration and follow-up obesity index disappeared for this group (β_BMI_ = 0.028, 95%CI_BMI_: -0.012 ~ 0.068; β_WC_ = 0.020, 95%CI_WC_: -0.022 ~ 0.062). Model fitting parameters were RMR = 0.007 and CFI = 1 in the Sleep duration-BMI model and RMR = 0.010 and CFI = 0.996 in the Sleep duration-WC model, indicating an acceptable model fitness.

### Sensitivity analyses

**Fig. 3:**
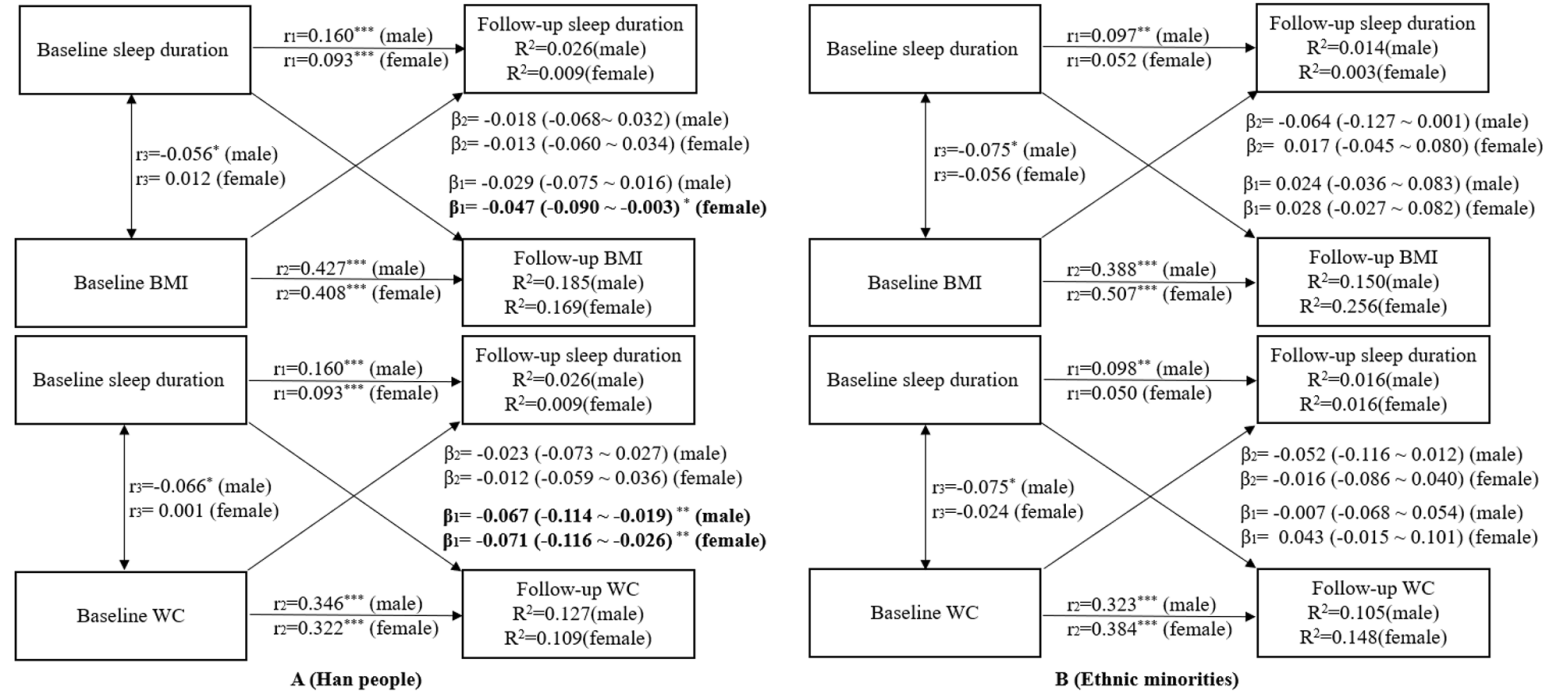
Cross-lagged path analysis of sleep duration with BMI and WC in the Han people (A) and Ethnic minorities (B) by sex groups, adjusted for age, place of residence, education levels, marital status, smoking status, alcohol consumption, dietary energy intake, physical activity, sedentary behavior, and follow-up years; β_1_ represents cross-lagged path coefficients from baseline sleep duration to follow-up BMI or WC; β_2_ represents from baseline BMI or WC to follow-up sleep duration; r_1_ and r_2_ represent tracking correlations; r_3_ represent synchronous correlations; R^2^ represents variance explained. Goodness-of-fit (A, Han people, BMI, male): CFI = 1, RMR = 0.004; Goodness-of-fit (A, Han people, BMI, female): CFI = 1, RMR = 0.003; Goodness-of-fit (A, Han people, WC, male): CFI = 0.989, RMR = 0.014; Goodness-of-fit (A, Han people, WC, female): CFI = 1, RMR = 0.006; Goodness-of-fit (B, Ethnic minorities, BMI, male): CFI = 1, RMR = 0.009; Goodness-of-fit (B, Ethnic minorities, BMI, female): CFI = 1, RMR = 0.003; Goodness-of-fit (B, Ethnic minorities, WC, male): CFI = 1, RMR = 0.007; Goodness-of-fit (B, Ethnic minorities, WC, female): CFI = 0.998, RMR = 0.011. The cross-lagged path coefficients are presented as β (lower 95% CI, upper 95% CI). ^*^P ≤ 0.05, ^**^P ≤ 0.01, ^***^P ≤ 0.001

We performed the first sensitivity analysis using bootstrap simulation and obtained 95% confidence intervals for cross-lagged path coefficients (**Supplement** Fig. 1). Though the confidence interval changed slightly, the results corroborated with the conclusion from cross-lagged path analyses. We performed the second sensitivity analysis by estimating the cross-lagged path coefficients for males and females separately. For Han people, the results showed a subtle gender difference in the relationship between sleep duration and follow up BMI, but not follow up WC. Precisely, the path coefficient from baseline sleep duration to follow-up BMI was significant in the Han people female (β_BMI_ = -0.047, 95%CI_BMI_: -0.090 ~ -0.003) but not significant in the Han people male (β_BMI_= -0.029, 95%CI_BMI_: -0.075 ~ 0.016) (Fig. 3A). For ethnic minorities, the results showed no significant relationship of sleep duration with BMI and WC, and this non-significant relationship did not differ by gender (Fig. 3B).

## Discussion

To the best of our knowledge, this study is the first to investigate the temporal relationship between sleep duration and obesity among Chinese Han people and Chinese ethnic minorities. The results showed that the relationship pattern between sleep duration and obesity across Han people and ethnic minorities was different.

For Han people, our results showed that sleep duration has an impact on subsequent obesity, but not vice versa. For the inverse effect of sleep duration on obesity, our results are consistent with previous findings in the general population [7, 8]. Previous studies have shown that sleep deprivation may decrease leptin and increase gastric hunger hormone levels, leading to increased appetite and increased food intake [32]. In parallel, sleep deprivation, as a metabolic stressor, may activate the hypothalamic-pituitary-adrenal (HPA) axis and increase cortisol production, thereby increasing food intake and leading to visceral fat accumulation [33, 34]. In addition, people who sleep less may be more fatigued, which may reduce physical activity and increase sedentary time, thereby leading to obesity [33]. These potential mechanisms explain, to some extent, the inverse association between sleep duration and subsequent obesity. However, for the insignificant effect of BMI and WC on sleep duration, our findings are not consistent with several prior studies [10, 11]. This difference in the inverse association may be due to differences in sleep duration measurements, adjustment for confounders, and characteristics of the participants.

Besides, For Han people, this study also found gender differences in the effect of sleep duration on BMI. Specifically, the negative relationship between baseline sleep duration and follow-up BMI was significant only in female, but not in male, which is consistent with previous findings [35, 36]. However, the exact biological mechanism for this gender difference is not clear. One possible explanation is that it is related to sexual hormones [37]. Previous studies have found that changes in sexual hormones not only lead to a decrease in sleep quality [38], but also to an increase in body weight [39]. Compared to males, females are more likely to suffer from changes in sexual hormones (such as menopause) and experience a decrease in sleep duration leading to obesity.

For ethnic minorities, our findings showed that there was no relationship between sleep duration and obesity at all. This is consistent with a few studies of ethnic minorities in the United States failing to find a link between sleep duration and obesity [40]. For this non-significant association in ethnic minorities, we argued that the relatively long sleep duration of ethnic minorities may play an important role. Previous studies have shown that there is no significant relationship between sleep duration and obesity for people with a long sleep duration [4]. From this prospective, our results did show that ethnic minorities had a longer sleep duration than Han people, thus the significant association disappearing. As to the reason why the ethnic minorities having a longer sleep duration, the socioeconomic status (SES) may be one of the key factors. Compared to Han people, most ethnic minorities live in remote and poor areas and have lower SES [20]. Previous studies indicated that lower SES is associated with longer sleep duration [41]. For instance, some studies found that living in a rural area and having a lower level of educational attainment are protective factors for long sleep duration [42, 43]. Thus, among ethnic minorities with lower SES can have a longer sleep duration, attributing to a non-significant association between sleep duration and obesity. Nevertheless, due to the lack of relevant studies on the relationship between sleep duration and obesity among Chinese ethnic minorities, further studies with large samples are still needed to confirm our findings.

There are several limitations to this study. First, participants self-reported sleep duration might not reflect the actual sleep duration. Objective measurements of sleep duration are worth considering in future studies. Moreover, information for other sleep-related characteristics, such as sleep quality, sleep patterns and sleep disorder, was missed due to the limitations of the dataset. The lack of considering these potential confounders may bring about a spurious link between sleep duration and obesity. In addition, the results of this study are based on data from Guizhou province, China. The generalization of the findings to the whole Chinese or global population may be limited.

## Conclusion

Our results suggested an ethnic difference in the relationship between sleep and obesity for Chinese populations. Given the backdrop of the obesity epidemic in China, future sleep-aimed overweight and obesity interventions should be conducted according to population characteristics.

## Electronic supplementary material

Below is the link to the electronic supplementary material.

Supplementary Material 1

## Data Availability

The data that support this study are available from the corresponding author upon reasonable request.

## Abbreviations

GPHCS: Guizhou Population Health Cohort Study
WC: Waist circumference
BMI: Body mass index
FFQ: Food frequency questionnaire
RMR: Root mean square residual
CFI: Comparative fit index
SES: Socioeconomic status

## References

[1] Bluher M (2019). Obesity: global epidemiology and pathogenesis. Nat Rev Endocrinol.

[2] Liu J, He Z, Ma N, Chen ZY (2020). Beneficial Effects of Dietary Polyphenols on High-Fat Diet-Induced obesity linking with modulation of gut microbiota. J Agric Food Chem.

[3] Garfield V. The Association between Body Mass Index (BMI) and sleep duration: where are we after nearly two decades of Epidemiological Research? Int J Environ Res Public Health. 2019;16(22). 10.3390/ijerph16224327. 10.3390/ijerph16224327 PMC6888565 31698817

[4] Zhou Q, Zhang M, Hu D (2019). Dose-response association between sleep duration and obesity risk: a systematic review and meta-analysis of prospective cohort studies. Sleep Breath.

[5] Bacaro V, Ballesio A, Cerolini S, Vacca M, Poggiogalle E, Donini LM (2020). Sleep duration and obesity in adulthood: an updated systematic review and meta-analysis. Obes Res Clin Pract.

[6] Guimaraes KC, Silva CM, Latorraca COC, Oliveira RA, Crispim CA (2022). Is self-reported short sleep duration associated with obesity? A systematic review and meta-analysis of cohort studies. Nutr Rev.

[7] Cho KH, Cho EH, Hur J, Shin D (2018). Association of Sleep duration and obesity according to gender and age in korean adults: results from the Korea National Health and Nutrition Examination Survey 2007–2015. J Korean Med Sci.

[8] Jefferson T, Addison C, Sharma M, Payton M, Jenkins BC (2019). Association between sleep and obesity in African Americans in the Jackson Heart Study. J Am Osteopath Assoc.

[9] Singh M, Drake CL, Roehrs T, Hudgel DW, Roth T (2005). The association between obesity and short sleep duration: a population-based study. J Clin Sleep Med.

[10] Garfield V, Llewellyn CH, Steptoe A, Kumari M (2017). Investigating the bidirectional Associations of Adiposity with Sleep Duration in older adults: the English Longitudinal Study of Ageing (ELSA). Sci Rep.

[11] Sokol RL, Grummon AH, Lytle LA (2020). Sleep duration and body mass: direction of the associations from adolescence to young adulthood. Int J Obes (Lond).

[12] Koolhaas CM, Kocevska D, Te Lindert BHW, Erler NS, Franco OH, Luik AI (2019). Objectively measured sleep and body mass index: a prospective bidirectional study in middle-aged and older adults. Sleep Med.

[13] Pan XF, Wang L, Pan A (2021). Epidemiology and determinants of obesity in China. Lancet Diabetes Endocrinol.

[14] Wang L, Zhou B, Zhao Z, Yang L, Zhang M, Jiang Y (2021). Body-mass index and obesity in urban and rural China: findings from consecutive nationally representative surveys during 2004-18. Lancet.

[15] Jin Y, Luo Y, He P (2019). Hypertension, socioeconomic status and depressive symptoms in chinese middle-aged and older adults: findings from the China health and retirement longitudinal study. J Affect Disord.

[16] Tong X, Wang X, Wang D, Chen D, Qi D, Zhang H (2019). Prevalence and ethnic pattern of overweight and obesity among middle-aged and elderly adults in China. Eur J Prev Cardiol.

[17] Lu WH, Zhang WQ, Sun F, Gao YT, Zhao YJ, Liu JW (2021). Correlation between occupational stress and Coronary Heart Disease in Northwestern China: a case study of Xinjiang. Biomed Res Int.

[18] Ning X, Lv J, Guo Y, Bian Z, Tan Y, Pei P (2020). Association of Sleep Duration with Weight Gain and General and central obesity risk in chinese adults: a prospective study. Obes (Silver Spring).

[19] Zhou Q, Wu X, Zhang D, Liu L, Wang J, Cheng R (2020). Age and sex differences in the association between sleep duration and general and abdominal obesity at 6-year follow-up: the rural chinese cohort study. Sleep Med.

[20] Wang YJ, Chen XP, Chen WJ, Zhang ZL, Zhou YP, Jia Z (2020). Ethnicity and health inequalities: an empirical study based on the 2010 China survey of social change (CSSC) in Western China. BMC Public Health.

[21] Huang CQ, Dong BR, Lu ZC, Yue JR, Liu QX (2010). Chronic diseases and risk for depression in old age: a meta-analysis of published literature. Ageing Res Rev.

[22] Chen Y, Wang Y, Xu K, Zhou J, Yu L, Wang N, et al. Adiposity and long-term Adiposity Change are Associated with Incident Diabetes: a prospective cohort study in Southwest China. Int J Environ Res Public Health. 2021;18(21). 10.3390/ijerph182111481. 10.3390/ijerph182111481 PMC8582792 34769995

[23] Zhang Y, Wang Y, Chen Y, Zhou J, Xu L, Xu K, et al. Associations of dietary patterns and risk of hypertension in Southwest China: a prospective cohort study. Int J Environ Res Public Health. 2021;18(23). 10.3390/ijerph182312378. 10.3390/ijerph182312378 PMC8656527 34886102

[24] Liu MJ, Li HT, Yu LX, Xu GS, Ge H, Wang LL, et al. A correlation study of DHA Dietary Intake and plasma, erythrocyte and breast milk DHA concentrations in Lactating Women from Coastland, Lakeland, and Inland Areas of China. Nutrients. 2016;8(5). 10.3390/nu8050312. 10.3390/nu8050312 PMC4882724 27213448

[25] Bull FC, Maslin TS, Armstrong T (2009). Global physical activity questionnaire (GPAQ): nine country reliability and validity study. J Phys Act Health.

[26] Hamrik Z, Sigmundova D, Kalman M, Pavelka J, Sigmund E (2014). Physical activity and sedentary behaviour in Czech adults: results from the GPAQ study. Eur J Sport Sci.

[27] Kivimaki M, Feldt T, Vahtera J, Nurmi JE (2000). Sense of coherence and health: evidence from two cross-lagged longitudinal samples. Soc Sci Med.

[28] Zhang T, Zhang H, Li Y, Sun D, Li S, Fernandez C (2016). Temporal relationship between Childhood Body Mass Index and insulin and its impact on adult hypertension: the Bogalusa Heart Study. Hypertension.

[29] Han T, Lan L, Qu R, Xu Q, Jiang R, Na L (2017). Temporal relationship between hyperuricemia and insulin resistance and its impact on future risk of hypertension. Hypertension.

[30] Rosseel Y (2012). lavaan: an R package for structural equation modeling. J Stat Softw.

[31] Joreskog KG (1996). Modeling development: using covariance structure models in longitudinal research. Eur Child Adolesc Psychiatry.

[32] Taheri S, Lin L, Austin D, Young T, Mignot E (2004). Short sleep duration is associated with reduced leptin, elevated ghrelin, and increased body mass index. PLoS Med.

[33] Kyrou I, Tsigos C (2009). Stress hormones: physiological stress and regulation of metabolism. Curr Opin Pharmacol.

[34] Magee CA, Huang XF, Iverson DC, Caputi P. Examining the pathways linking chronic sleep restriction to obesity. *J Obes* 2010, 2010. 10.1155/2010/821710. 10.1155/2010/821710 PMC2925323 20798899

[35] Cournot M, Ruidavets JB, Marquie JC, Esquirol Y, Baracat B, Ferrieres J (2004). Environmental factors associated with body mass index in a population of Southern France. Eur J Cardiovasc Prev Rehabil.

[36] Ogilvie RP, Bazzano LA, Gustat J, Harville EW, Chen W, Patel SR (2019). Sex and race differences in the association between sleep duration and adiposity: the Bogalusa Heart Study. Sleep Health.

[37] Meyer KA, Wall MM, Larson NI, Laska MN, Neumark-Sztainer D (2012). Sleep duration and BMI in a sample of young adults. Obes (Silver Spring).

[38] Sowers MF, Zheng H, Kravitz HM, Matthews K, Bromberger JT, Gold EB (2008). Sex steroid hormone profiles are related to sleep measures from polysomnography and the Pittsburgh Sleep Quality Index. Sleep.

[39] Lovejoy JC (1998). The influence of sex hormones on obesity across the female life span. J Womens Health.

[40] Sun X, Gustat J, Bertisch SM, Redline S, Bazzano L (2020). The association between sleep chronotype and obesity among black and white participants of the Bogalusa Heart Study. Chronobiol Int.

[41] Patel SR, Malhotra A, Gottlieb DJ, White DP, Hu FB (2006). Correlates of long sleep duration. Sleep.

[42] Ren Y, Liu Y, Meng T, Liu W, Qiao Y, Gu Y (2019). Social-biological influences on sleep duration among adult residents of northeastern China. Health Qual Life Outcomes.

[43] Wang S, Li B, Wu Y, Ungvari GS, Ng CH, Fu Y (2017). Relationship of Sleep Duration with Sociodemographic characteristics, Lifestyle, Mental Health, and chronic Diseases in a large Chinese Adult Population. J Clin Sleep Med.

