# Supplementary material for: Temporal relationship between sleep duration and obesity among Chinese Han people and ethnic minorities
Source: BMC Public Health. 2023 Mar 15;23:503. doi: 10.1186/s12889-023-15413-4 (PMC10015728; doi:10.1186/s12889-023-15413-4)
Supplement: Supplementary file 1 — Supplementary Material 1 [file 12889_2023_15413_MOESM1_ESM.docx]

**Appendix. Supplementary**

**Temporal Relationship Between Sleep Duration and Obesity Among Chinese Han people and** **Ethnic Minorities**

Zhengxing Xu, Min Chen, Yuntong Yao, Lisha Yu, Peijing Yan, Huijie Cui, Ping Li, Jiaqiang Liao, Ben Zhang, Yuqin Yao, Zhenmi Liu, Xia Jiang, Tao Liu, Chenghan Xiao

**Corresponding authors:**

Tao Liu, E-mail: liutaombs@163.com

Chenghan Xiao, E-mail: [chenghan_xiao@scu.edu.cn](mailto:chenghan_xiao@scu.edu.cn)

Supplement Table 1. Baseline characteristics of included and excluded participants

|  | Included participants | Excluded participants | *P-*value |
| --- | --- | --- | --- |
| Age, years | 44.02±14.95 | 43.84±15.37 | 0.617 |
| Male | 2,425 (47.59) | 1,447 (47.18) | 0.722 |
| Han people | 3,188 (62.56) | 1,814 (59.15) | 0.002 |
| Urban | 1,796 (35.24) | 922 (30.06) | <0.001 |
| Education |  |  | <0.001 |
| Illiteracy | 1,036 (20.33) | 651 (21.23) |  |
| Primary | 1,734 (34.03) | 1,258 (41.02) |  |
| Secondary | 2,050 (40.23) | 1,076 (35.08) |  |
| Collage and above | 276 (5.42) | 82 (2.67) |  |
| Married | 4,099 (80.44) | 2,460 (80.21) | 0.803 |
| Smoking | 1,480 (29.04) | 856 (27.91) | 0.273 |
| Drinking | 1,694 (33.24) | 928 (30.33) | 0.006 |
| Energy intake, kcal/d | 2,112.09±855.57 | 2,158.14±835.38 | 0.020 |
| Physical activity |  |  | <0.001 |
| Low | 1,289 (25.29) | 698 (23.30) |  |
| Moderate | 1,126 (22.10) | 502 (16.76) |  |
| High | 2,681 (52.61) | 1,796 (59.95) |  |
| Sedentary duration, h | 4.09±2.25 | 3.99±2.11 | 0.041 |

Abbreviation: BMI = body mass index, WC = waist circumference.

Data are frequency (%) for categorical variables and mean ± standard deviation for continuous variables.

Supplement Table 2. Pearson Correlation Coefficients of the Z-transformed

|  | Variables | Baseline  sleep duration | Baseline BMI | Baseline WC | Follow-up sleep duration | Follow-up BMI | Follow-up WC |
| --- | --- | --- | --- | --- | --- | --- | --- |
| Han people | Baseline sleep duration | 1 |  |  |  |  |  |
|  | Baseline BMI | -0.023 | 1 |  |  |  |  |
|  | Baseline WC | -0.038^*^ | 0.734^***^ | 1 |  |  |  |
|  | Follow-up sleep duration | 0.126^***^ | -0.019 | -0.023 | 1 |  |  |
|  | Follow-up BMI | -0.051^**^ | 0.421^***^ | 0.343^***^ | -0.026 | 1 |  |
|  | Follow-up WC | -0.083^***^ | 0.339^***^ | 0.345^***^ | -0.050^**^ | 0.718^***^ | 1 |
| Ethnic minorities | Baseline sleep duration | 1 |  |  |  |  |  |
|  | Baseline BMI | -0.064^**^ | 1 |  |  |  |  |
|  | Baseline WC | -0.047^*^ | 0.756^***^ | 1 |  |  |  |
|  | Follow-up sleep duration | 0.073^**^ | -0.027 | -0.046^*^ | 1 |  |  |
|  | Follow-up BMI | -0.001 | 0.453^***^ | 0.365^***^ | 0.012 | 1 |  |
|  | Follow-up WC | 0.003 | 0.343^***^ | 0.360^***^ | 0.015 | 0.689^***^ | 1 |

Abbreviation: BMI = body mass index, WC = waist circumference.

^*^ *P* ≤ 0.05, ^**^ *P* ≤ 0.01, ^***^ *P* ≤ 0.001.


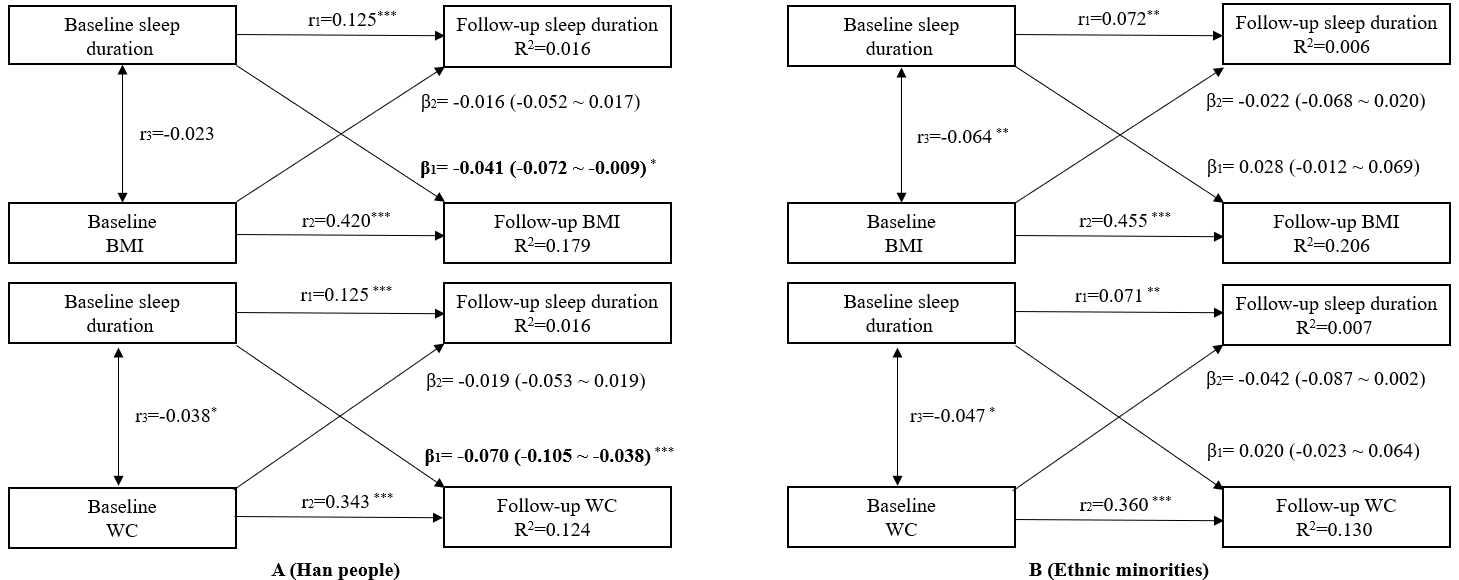


**Supplement Figure 1** Cross-lagged path analysis of sleep duration with BMI and WC in the Han people (A) and ethnic minorities (B) by bootstrap simulation, adjusted for age, gender, place of residence, education levels, marital status, smoking status, alcohol consumption, dietary energy intake, physical activity, sedentary behavior, and follow-up years; β_1_ represents cross-lagged path coefficients from baseline sleep duration to follow-up BMI or WC; β_2_ represents from baseline BMI or WC to follow-up sleep duration; r_1_ and r_2_ represent tracking correlations; r_3_ represent synchronous correlations; R^2^ represents variance explained. Goodness-of-fit (A, Han people, BMI): CFI=1, RMR=0.004; Goodness-of-fit (A, Han people, WC): CFI=0.994, RMR=0.010; Goodness-of-fit (B, Ethnic minorities, BMI): CFI=1, RMR=0.007; Goodness-of-fit (B, Ethnic minorities, WC): CFI=0.996, RMR=0.010. The cross-lagged path coefficients are presented as β (lower 95% CI, upper 95% CI). ^*^ *P* ≤ 0.05, ^**^ *P* ≤ 0.01, ^***^ *P* ≤ 0.001.
